# Free Triiodothyronine Is Associated With Hepatic Steatosis and Liver Stiffness in Euthyroid Chinese Adults With Non-Alcoholic Fatty Liver Disease

**DOI:** 10.3389/fendo.2021.711956

**Published:** 2021-08-12

**Authors:** Wen Guo, Pei Qin, Xiao-Na Li, Juan Wu, Jing Lu, Wen-Fang Zhu, Qing-qing Diao, Nian-Zhen Xu, Qun Zhang

**Affiliations:** Department of Health Promotion Center, The First Affiliated Hospital With Nanjing Medical University, Nanjing, China

**Keywords:** free triiodothyronine, hepatic steatosis, liver stiffness, non-alcoholic fatty liver disease, euthyroidism

## Abstract

**Objective:**

The association between non-alcoholic fatty liver disease (NAFLD) and thyroid hormones in euthyroid subjects is unclear. We investigated the relationship between thyroid function and the severity of hepatic steatosis and liver fibrosis in a large cohort of euthyroid Chinese adults.

**Methods:**

A total of 3496 participants were enrolled. Liver ultrasonography was used to define the presence of NAFLD (n=2172) or the absence of NAFLD (n=1324). Anthropometric and biochemical measurements were made and thyroid function parameters including free triiodothyronine (FT3), free thyroxine (FT4), thyroid‐stimulating hormone (TSH) were measured. The severity of hepatic steatosis and liver stiffness was assessed by transient elastography.

**Results:**

Levels of FT3 were significantly higher in the severe NAFLD group and moderate NAFLD group than in the mild NAFLD group (5.18 ± 0.58 *vs* 5.11 ± 0.57 *vs* 4.98 ± 0.60 pmol/L, *P*<0.001). Participants with F4 and F3 liver fibrosis had higher FT3 levels than those with F2 fibrosis (6.33 ± 0.39 *vs* 5.29 ± 0.48 *vs* 5.20 ± 0.50 pmol/L, *P*<0.001). However, FT4 and TSH levels did not correlate with hepatic steatosis or liver fibrosis severity. In addition, the proportions of participants with NAFLD (46.0% *vs* 63.1% *vs* 73.3%, *P*<0.001) and liver fibrosis (11.5% *vs* 18.6% *vs* 20.8%, *P*<0.001) increased as FT3 levels increased. Logistic regression analysis showed that FT3 levels were positively associated with the severity of hepatic steatosis and liver fibrosis presence, even after adjustment for metabolic risk factors including BMI. In non-obese participants, the FT3 level was an independently risk factor for the severity of hepatic steatosis.

**Conclusions:**

There are positive associations of FT3 levels with the severity of hepatic steatosis and the presence of liver fibrosis in NAFLD with euthyroidism.

## Introduction

Non-alcoholic fatty liver disease (NAFLD) is a wide-spectrum liver disease, which will evolve to non-alcoholic steatohepatitis (NASH), liver fibrosis, and cirrhosis. With improvement of people’s living standard and rapid lifestyle transitions, the prevalence of NAFLD in China is 29.2% (1), and by 2030 China will have the highest growth in the prevalence of NAFLD globally, with 315 million cases (2), representing a substantial clinical burden and a public health concern. Because there are no obvious symptoms in the early stages of NAFLD, this problem can be invisible for patients and doctors for years. Besides the leading cause of chronic liver disease, more recent evidence suggested that NAFLD was closely associated with metabolic syndrome, type 2 diabetes and cardiovascular disease (CVD) (3–5). Although understanding regarding management and treatment of its risk factors (e.g. diabetes and dyslipidemia) has improved substantially, the prevalence of NAFLD has rapidly increased. Hence, investigation of additional modifiable risk factors is urgently needed.

Because of the importance of thyroid hormones in lipid metabolism and energy homeostasis, which participate in the development and progression of NAFLD, the relationship between thyroid diseases and NAFLD has attracted close attention, yet conclusions from studies investigating the relationship between thyroid diseases and NAFLD are inconsistent, varying from a strong association to no association (6–8). Meanwhile, conclusions regarding the association of free triiodothyronine (FT3), free thyroxine (FT4), and thyroid‐stimulating hormone (TSH) with NAFLD in euthyroid individuals remain inconsistent. For example, a study in the middle-aged and elderly euthyroid subjects showed that high-normal FT3 and low-normal TSH are independently associated with a higher incidence of NAFLD, but not by FT4 (9). The Lifelines Cohort Study indicated that NAFLD was independently associated with a high‐normal FT3 level, but not by TSH (10). There are limited studies, which aimed to assess the association of liver fibrosis with thyroid function within the euthyroid range in NAFLD.

Liver biopsy is the gold standard for the diagnosis of NAFLD and NAFLD -related advanced fibrosis, but its clinical application is limited due to its invasive and expensive. A number of noninvasive biomarkers of NAFLD severity and liver fibrosis are also being developed to identify those likely to have progressive liver disease or complications, such as transient elastography (11). The present study aimed to explore the relationship between thyroid function and the severity of hepatic steatosis/liver fibrosis evaluated by transient elastography in a large cohort of euthyroid Chinese population. On the other hand, obesity can affect thyroid function, subsequently alter its functional status in target organs and body metabolism (12). Therefore, another aim of the present study is to explore whether obesity plays a role in the association between thyroid function and the severity of hepatic steatosis/liver fibrosis in euthyroid.

## Materials and Methods

### Study Population

Participants were enrolled from the Health Promotion Center of the First Affiliated Hospital of Nanjing Medical University for health examinations between November 2017 and December 2019. They were further divided into the NAFLD group (n =2172) and the non-NAFLD group (n =1324) according to the results of liver ultrasonography. Written consents were obtained from all participants. Exclusion criteria were:1) excessive alcohol use (>140g/week for men and >70 g/week for women); 2) previously diagnosed hepatitis, cirrhosis or other chronic liver diseases (e.g., autoimmune liver disease, primary sclerosing cholangitis, drug-induced liver disease); 3) history of thyroidectomy, overt thyroid dysfunction, autoimmune thyroiditis, or thyroid tumor; 4) previous or current use thyroid hormone or anti‐thyroid drugs, or drugs that cause a fatty liver and/or liver fibrosis; 5) previously diagnosed chronic kidney disease, stroke, acute coronary syndrome, malignant tumors. The study protocol was approved by the Human Research Ethics Committee of the First Affiliated Hospital of Nanjing Medical University.

### Physical Examination and Biochemical Tests

Weight, height and blood pressure were measured in accordance with international standards. Venous blood samples were collected after an overnight fast. Routine biochemical analyses including measurement of serum lipids, glucose, hepatic function and uric acid, by enzymatic methods (Chemistry Analyzer AU5800, Olympus Medical Engineering Company, Japan). Thyroid function (FT3, FT4 and TSH) was determined by electro chemiluminescent methods (Roche Diagnostics GmbH, Mannheim, Germany). Glycated hemoglobin A1c (HbA1c) levels were measured by high‐performance liquid chromatography. Liver ultrasonography was performed in all participants.

### Liver Stiffness and Steatosis

Transient elastography was measured in participants of the NAFLD group in order to evaluate the hepatic steatosis severity and the presence of fibrosis. Transient elastography was performed using FibroTouch (Wuxi Hayes Kell Medical Technology Co. Ltd., China) system according to the operations manual. FibroTouch is a newly developed device for the evaluation of hepatic steatosis and fibrosis that is comparable with that of the FibroScan (13). Liver fibrosis and hepatic steatosis were determined using liver stiffness measurement (LSM) and the fat attenuation parameter (FAP), respectively.

### Statistical Analysis

Continuous variables were expressed as mean ± SD. Intergroup comparisons were done using Student’s t test or one-way ANOVA with Bonferroni correction for multiple comparisons. Categorical variables were expressed as percentages (numbers) and analysed by Pearson’s chi-squared test. Binary or multinomial logistic regression analysis was performed to investigate the independent predictors of NAFLD and liver fibrosis. Data were analysed using SPSS 18.0 statistical software, with significance defined as *P*<0.05 (two-sided).

## Results

### Baseline Characteristics of the Study Population

3496 participants were divided into the NAFLD group (n =2172) and the non-NAFLD group (n =1324) according to the results of liver ultrasonography. Clinical, demographic and biochemical characteristics of the participants are listed in **Table 1**. Compared to those without NAFLD, patients with NAFLD were more likely to be male, and had higher body mass index (BMI), systolic blood pressure (SBP), diastolic blood pressure (DBP), fasting blood glucose (FBG), HbA1c, serum uric acid, ALT, AST, GGT, FT3, total cholesterol (TC), triglyceride (TG), and low-density lipoprotein cholesterol (LDL-C) levels, yet lower levels of high-density lipoprotein cholesterol (HDL-C) (all *P*<0.05). There were no differences in age, FT4 and TSH levels between two groups.

**Table 1:** Baseline characteristics of individuals with or without NAFLD.

	Non‐NAFLD (n = 1324)	NAFLD (n = 2172)
Age (years)	49.28 ± 10.46	49.49 ± 9.86
Sex (male/female)	669/655	1755/417^*^
BMI (kg/m2)	22.88 ± 2.20	26.78 ± 2.59^*^
SBP (mmHg)	122.37 ± 17.14	130.45 ± 15.96^*^
DBP (mmHg)	74.51 ± 11.03	81.13 ± 10.92^*^
FBG (mmol/L)	5.26 ± 0.91	5.77 ± 1.44^*^
HbA1c (%)	5.53 ± 0.57	5.78 ± 0.85^*^
TC (mmol/L)	5.27 ± 1.04	5.41 ± 1.08^*^
TG (mmol/L)	1.32 ± 0.68	2.25 ± 1.64^*^
LDL-C(mmol/L)	3.22 ± 0.76	3.42 ± 0.77^*^
HDL-C(mmol/L)	1.43 ± 0.32	1.21 ± 0.25^*^
Uric acid (mmol/l)	311.08 ± 80.52	376.63 ± 83.21^*^
ALT (U/L)	19.39 ± 10.43	30.93 ± 19.59^*^
AST (U/L)	22.11 ± 7.21	25.39 ± 10.26^*^
GGT (U/L)	25.57 ± 19.82	43.59 ± 28.96^*^
FT3 (pmol/L)	4.77 ± 0.48	5.08 ± 0.59^*^
FT4 (pmol/L)	16.96 ± 1.98	17.06 ± 1.95
TSH (pmol/L)	2.25 ± 0.83	2.20 ± 0.84

Values are presented as mean ± standard deviation.

BMI, body mass index; SBP, systolic blood pressure; DBP, diastolic blood pressure; FBG, fasting blood glucose; TC, total cholesterol; TG, triacylglyceride; LDL-C, high-density lipoprotein cholesterol; HDL-C, high-density lipoprotein cholesterol; ALT, alanine aminotransferase; AST, aspartate transaminase; GGT, gamma-glutamyl transpeptidase; FT3, free triiodothyronine; FT4, free thyroxine; TSH, thyroid‐stimulating hormone. Compared with non‐NAFLD, ^*^P < 0.05.

### FT3 but Not FT4 and TSH Levels Are Associated With Hepatic Steatosis

**Figure 1:**
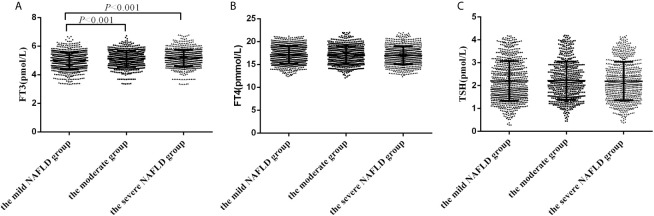
The levels of FT3, FT4 and TSH in subjects with different severity of hepatic steatosis of NAFLD. **(A)** The levels of FT3 in subjects with different severity of hepatic steatosis of NAFLD; **(B)** The levels of FT4 in subjects with different severity of hepatic steatosis of NAFLD; **(C)** The levels of TSH in subjects with different severity of hepatic steatosis of NAFLD.

2172 participants with NAFLD were further divided into the mild NAFLD(n=803), moderate NAFLD (n=597) and severe NAFLD(n=772) according to the value of FAP using Transient elastography. Patients in the severe NAFLD group and moderate NAFLD group had higher FT3 levels than those in the mild NAFLD (5.18 ± 0.58 *vs* 5.11 ± 0.57 *vs* 4.98 ± 0.60 pmol/L, *P*<0.001). However, no statistically significant differences in FT4 and TSH levels were detected among groups (**Figures 1A–C**).

### Baseline Characteristics of Individuals With or Without Liver Fibrosis in NAFLD

Participants with NAFLD were also further divided into four groups according to the value of LSM: F0-F1 (LSM ≤ 7.3kPa, n=1804), F2 (7.3 < LSM ≤ 9.7 kPa, n=245), F3 (9.7<LSM ≤ 12.4kPa, n=91), F4 (LSM > 12.4kPa, n=32). According to the operations manual, participants in the F0-F1 stage were recognised as non-liver fibrosis and participants in the F2-F4 stage were recognised as liver fibrosis. Clinical and biochemical characteristics of individuals with or without liver fibrosis in NAFLD are listed in **Table 2**. Compared to those without liver fibrosis, patients with liver fibrosis more likely to be male. In addition, subjects with liver fibrosis had higher BMI, SBP, DBP, FBG, HbA1c, TG, serum uric acid, ALT, AST, GGT and FT3 level than those without liver fibrosis (all *P*<0.05). There were no differences of TC, LDL-C, FT4 and TSH levels between two groups.

**Table 2:** Baseline characteristics of individuals with or without liver fibrosis in NAFLD.

	Non-liver fibrosis (n=1804)	liver fibrosis(n=368)
Age (years)	49.34 ± 9.84	50.26 ± 9.93
Sex (male/female)	1433/371	322/46^*^
BMI (kg/m2)	26.51 ± 2.48	28.07 ± 2.69^*^
SBP (mmHg)	129.67 ± 15.73	134.31 ± 16.53^*^
DBP (mmHg)	80.68 ± 10.92	83.30 ± 10.67^*^
FBG (mmol/L)	5.69 ± 1.36	6.16 ± 1.74^*^
HbA1c (%)	5.73 ± 0.79	6.02 ± 1.03^*^
TC (mmol/L)	5.41 ± 1.09	5.44 ± 1.06
TG (mmol/L)	2.20 ± 1.55	2.50 ± 2.02^*^
LDL-C(mmol/L)	3.42 ± 0.77	3.43 ± 0.76
HDL-C(mmol/L)	1.21 ± 0.25	1.17 ± 0.27^*^
Uric acid (mmol/l)	374.27 ± 83.46	388.14 ± 81.12^*^
ALT (U/L)	29.50 ± 18.02	37.97 ± 24.81^*^
AST (U/L)	24.68 ± 9.27	28.83 ± 13.64^*^
GGT (U/L)	42.34 ± 28.39	49.72 ± 30.90^*^
FT3 (pmol/L)	5.03 ± 0.58	5.32 ± 0.58^*^
FT4 (pmol/L)	17.04 ± 1.94	17.14 ± 2.01
TSH (pmol/L)	2.20 ± 0.85	2.21 ± 0.82

Values are presented as mean ± standard deviation.

BMI, body mass index; SBP, systolic blood pressure; DBP, diastolic blood pressure; FBG, fasting blood glucose; TC, total cholesterol; TG, triacylglyceride; LDL-C, high-density lipoprotein cholesterol; HDL-C, high-density lipoprotein cholesterol; ALT, alanine aminotransferase; AST, aspartate transaminase; GGT, gamma-glutamyl transpeptidase; FT3, free triiodothyronine; FT4, free thyroxine; TSH, thyroid‐stimulating hormone. Compared with non-liver fibrosis, ^*^P < 0.05.

### Comparison of FT3, FT4, and TSH Levels in Different Fibrosis Stages in Participants With Liver Fibrosis

**Figure 2:**
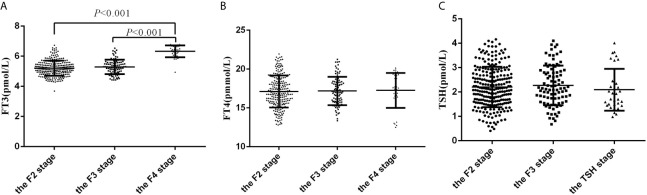
The levels of FT3, FT4 and TSH in subjects with F2, F3 and F4 fibrosis. **(A)** The levels of FT3 in subjects with F2, F3 and F4 fibrosis; **(B)** The levels of FT4 in subjects with F2, F3 and F4 fibrosis; **(C)** The levels of TSH in subjects with F2, F3 and F4 fibrosis.

Participants in the F4 stage and F3 stage had higher FT3 levels than those in F2 stage (6.33 ± 0.39 *vs* 5.29 ± 0.48 *vs* 5.20 ± 0.50 pmol/L, *P*<0.001). However, no statistically significant differences in FT4 and TSH levels were observed among the groups (**Figures 2A–C**).

### The Proportions of Participants With NAFLD and Liver Fibrosis Compared Across the Tertiles of FT3 Level

To further explore the relationship between FT3 level and NAFLD, all participants were stratified into tertiles of FT3 levels (tertiles1 <4.74pmol/L, n=1179; tertiles2 4.74-5.22pmol/L, n=1137; and tertiles3 >5.22pmol/L, n=1180), and the proportion of participants with NAFLD in different FT3 level tertiles was assessed. A continuous rise in the proportion of participants with NAFLD was observed along the tertiles of FT3 level (46.0% *vs* 63.1% *vs* 73.3%, *P*<0.001). Participants with NAFLD were stratified into three groups by the tertiles of FT3 level (tertiles 1<4.86pmol/L, n=730; tertiles2 4.86-5.34pmol/L, n =740; and tertiles3>5.34pmol/L, n=702). The proportion of participants with liver fibrosis was higher in the highest FT3 tertiles than in the middle and the lowest tertiles (11.5% *vs*18.6%*vs* 20.8%, *P*<0.001).

### Association of FT3 Level With the Severity Hepatic Steatosis and Liver Fibrosis by Logistic Regression Analysis

Multinomial logistic regression analyses were used to identify the association between FT3 level and hepatic steatosis severity. Clinically significant and statistically significant parameters from univariate analysis were included. The FT3 level was positively correlated with the mild NAFLD (*OR*=2004, 95%*CI* 1.702-2.360, *P*<0.001), moderate NAFLD (*OR*=3.110, 95%*CI* 2.584-3.744, *P*<0.001) and severe NAFLD (*OR*=3.919, 95%*CI* 3.288-4.670, *P*<0.001) without adjustment. After adjusting for sex, BMI, SBP, DBP, FBG, HbA1c, TC, TG, LDL-C, HDL-C and uric acid, the FT3 level was still positively correlated with mild NAFLD (*OR*=1.357, 95%*CI* 1.098-1.677, *P*=0.005), moderate NAFLD (*OR*=1.352, 95%*CI* 1.727-2.199, *P*<0.001) and severe NAFLD (*OR*=1.947, 95%*CI* 1.513-2.504, *P*<0.001). According to binary logistic regression analysis, the FT3 level was still positively correlated with liver fibrosis (*OR*=2.191, 95%*CI* 1.743-2.755, *P*<0.001) after adjusting for sex, BMI, SBP, DBP, FBG, HbA1c, TG, HDL-C and uric acid.

### Association of FT3 Level With the Severity Hepatic Steatosis and Liver Fibrosis by Logistic Regression Analysis in Non-Obese NAFLD

3496 participants were divided by their BMI into obese (BMI ≥25 kg/m^2^, n=2071) and non-obese (BMI <25 kg/m^2^, n=1425). Non-obese participants were divided the non-obese NAFLD(n=403) and the control group(n=1022) according to the results of liver ultrasonography. Non-obese NAFLD patients were further divided into the non-obese mild NAFLD(n=223), non-obese moderate NAFLD (n=111) and non-obese severe NAFLD(n=69) according to the value of FAP using Transient elastography. In non-obese participants, FT3 levels were significantly increased with the increase in the severity of hepatic steatosis (4.73 ± 0.48 *vs* 4.87 ± 0.64 *vs* 4.98 ± 0.58 *vs* 5.03 ± 0.57 pmol/L, *P*<0.01). However, no statistically significant differences in FT4 and TSH levels were observed among the groups. Multinomial logistic regression analysis showed that FT3 level was independently correlated with non-obese mild NAFLD (*OR*=1.323, 95%*CI* 1.053-1.838, *P*=0.045), non-obese moderate NAFLD (*OR*=1.609, 95%*CI* 1.024-2.528, *P*=0.039) and non-obese severe NAFLD (*OR*=2.288, 95%*CI* 1.320-3.964, *P*=0.003), even adjustment for BMI.

Participants with non-obese NAFLD were also further divided into the liver fibrosis group(n=26) and the non- liver fibrosis(n=377) according to the value of LSM. The levels of FT3 in patients in the liver fibrosis were higher compared to those without liver fibrosis (4.91 ± 0.61 *vs* 5.15 ± 0.62 pmol/L, *P*=0.06), but the difference was not statistically significant.

### Association of FT3 Level With the Severity Hepatic Steatosis and Liver Fibrosis by Logistic Regression Analysis in Obese NAFLD

Obese participants were divided the obese NAFLD group (n=1769) and obese without NAFLD group (n=302) according to the results of liver ultrasonography. Obese NAFLD patients were further divided into the obese mild NAFLD(n=580), obese moderate NAFLD (n=486) and obese severe NAFLD(n=703) according to the value of FAP using Transient elastography. In obese participants, FT3 levels were significantly increased with the increase in the severity of hepatic steatosis (4.87 ± 0.44 *vs* 5.02 ± 0.57 *vs* 5.14 ± 0.56 *vs* 5.19 ± 0.57 pmol/L, *P*<0.01). However, no statistically significant differences in FT4 and TSH levels were observed among the groups. In obese NAFLD, FT3 level was independently correlated with mild NAFLD (*OR*=1.374, 95%*CI* 1.021-1.849, *P*=0.036), moderate NAFLD (*OR*=1.754, 95%*CI* 1.273-2.418, *P*=0.001) and severe NAFLD (*OR*=1.894, 95%*CI* 1.367-2.624, *P*<0.001) after adjusted for confounding factors including BMI.

Patients with obese NAFLD were also further divided into the liver fibrosis group(n=342) and the non-liver fibrosis(n=1427) according to the value of LSM. Patients in the liver fibrosis group had higher FT3 levels than those in the non- liver fibrosis (5.07 ± 0.56 *vs* 5.34 ± 0.57 pmol/L, *P*<0.01). Binary logistic regression analysis showed that the FT3 level was still positively correlated with liver fibrosis (*OR*=2.415, 95%*CI* 1.882-3.097, *P*<0.001) in obese NAFLD, even adjusting for BMI.

## Discussion

Here for the first time, we demonstrate a relationship between thyroid function and the severity of hepatic steatosis and liver fibrosis evaluated by transient elastography in a large cohort of the euthyroid Chinese population. The major finding of our study was that FT3 level was positively associated with hepatic steatosis severity and the presence of liver fibrosis of NAFLD in euthyroid participants, independently of well-known metabolic risk factors including BMI.

The thyroid gland produces hormones that regulate energy metabolism, lipid and carbohydrate metabolism, and nervous system development. Knowledge regarding the association between thyroid function and risk of NAFLD is increasing, with evidence suggesting that thyroid dysfunction is associated with weight gain and development of metabolic dysfunction, including insulin resistance and lipid metabolism disorders (14, 15). Recently, these associations between thyroid hormones and NAFLD have been extended to euthyroid individuals by several studies, but with somewhat inconsistent results. For FT4, some studies showed that its concentrations were negatively associated with the risk for NAFLD (16, 17), while a cross-sectional study in euthyroid population indicated that the FT4 level was not significantly different between subjects with and without NAFLD (18). For TSH, inconsistent associations of serum TSH with NAFLD were reported with positive results or null results (10, 17, 19). Two Chinese studies and the Lifelines Cohort Study indicated that high‐normal FT3 levels were positively correlated with NAFLD, where the latter study used the fatty liver index to identify NAFLD (10, 20, 21). Another Chinese study showed that FT3 levels were inversely correlated with NAFLD (17). All these studies did not explore the association between thyroid function and the severity hepatic steatosis evaluated by transient elastography, especially in a large euthyroid population with NAFLD. In the present study, we found that the FT3 level was positively correlated with the severity of hepatic steatosis, even after adjusting for metabolic risk factors. Meanwhile, the proportion of participants with NAFLD was significantly increased along with the increasing levels of the FT3 level. But we did not find the association of the hepatic steatosis severity with the FT4 and TSH level. Although the exact mechanisms for the positive correlation between FT3 levels and hepatic steatosis severity are unclear but may involve iodothyronine deiodinase (DIO) expression. Previous studies showed that type 1 DIO (DIO1) activity was increased in the adipose tissue of obese humans and animals with a high‐fat diet (22, 23), hereby resulting in higher conversion of FT4 to FT3. We also found that FT3/FT4 ratios were significantly increased with the increase in the severity of hepatic steatosis (0.28 ± 0.03 *vs* 0.29 ± 0.04 *vs* 0.30 ± 0.04 *vs* 0.31 ± 0.04, *P*<0.01). Taken together, it is hypothesized that the increase in FT3 levels under unfavorable metabolic situations such as NAFLD might be a compensatory mechanism for fat accumulation. On the other hand, multiple pathways involved in lipid metabolism are affected by higher FT3 levels, including enhanced lipolysis in adipose tissue, increased the delivery of nonesterified fatty acids to the liver, and stimulated *de novo* lipogenesis in the liver (24, 25), and hence is probably involved in the pathogenesis of NAFLD. Clinical evidence showed that in euthyroid patients, higher FT3 was correlated with unfavorable metabolic profiles, including higher TG, fasting plasma glucose and insulin resistance (26, 27), which were all risk factors for NAFLD. However, it is still unclear whether changes in FT3 levels cause NAFLD or are a consequence of the disease. The relationship between FT3 and the severity of hepatic steatosis needs to be prospectively delineated in the future.

More recent some studies have focused on the correlation between FT3 levels with liver fibrosis in NAFLD subjects but yielded inconsistent results. In Patients with T2DM and NAFLD, a low FT3 level was an independent risk factor of advanced fibrosis, where this study used NAFLD fibrosis scores to identify advanced fibrosis (28). Zhang X et al. showed that the FT3 level did not predict an increased risk of advanced fibrosis in euthyroid Chinese population with NAFLD (17). Manka et al. (29) determined that low FT3, but not TSH and FT4, were associated with increased liver stiffness and NAFLD fibrosis scores, respectively. In contrast to the studies of Manka et al. and Zhang X et al., our study found that the FT3 level was independently correlated with liver fibrosis evaluated by transient elastography. In addition, the proportion of participants with liver fibrosis was significantly increased along with the increasing levels of FT3. A similar finding was observed by Liu et al. (18), who found that high FT3 levels were independently associated with fibrosis risk estimated by a BARD score ≥ 2 in patients with NAFLD. However, the study from Manka et al. is not directly comparable to our study because substantially more subjects in the Manka et al. study had significant liver fibrosis (Manka et al.: 66/144 (45.83%) > 7.6 kPa; current study: 386/2215 (17.42%) > 7.3 kPa. The study of Zhang X et al. used FIB-4 index ≥ 1.45 as a suitable noninvasive score to discriminate advanced hepatic fibrosis. Transient elastography was measured in NAFLD participants to evaluate liver fibrosis in the present study. It therefore seems plausible that the discrepancies may be related to the different study populations and diagnostic criteria for NAFLD-related advanced fibrosis. Future research with a prospective longitudinal study design and the larger sample size is needed to more definitively characterize the relationship between FT3 levels and liver fibrosis.

It is increasingly recognized that thyroid hormone levels are associated with effects on body fat (30). Euthyroid obese individuals had higher FT3 levels (31), while FT3 levels decreased after weight loss (32). Because obesity in the pathogenesis of NAFLD is well established, it may be a confounding factor of the relationship between FT3 levels and NAFLD. We adjusted BMI and found that the positive associations of the FT3 level with hepatic steatosis severity and the presence of liver fibrosis of NAFLD still existed. In addition, all participants in the present were divided into the obese and non-obese group according to their BMI. We still found that high-normal FT3 level was an independently risk factor for the severity of hepatic steatosis in non-obese participants. Unfortunately, we did not find the relationship between the FT3 level and liver fibrosis in non-obese NAFLD due to the small sample size of the liver fibrosis group. We speculated that obesity did not participant in the association between the FT3 level and the severity of hepatic steatosis. Clearly, the cross-sectional design of the present study hampers to establish a sequence of metabolic changes, and the possible interrelationship of obesity with the development of NAFLD needs to be prospectively delineated in future.

This study has several limitations. First, the cross-sectional design means it is difficult to determine whether FT3 levels have a causative effect on the severity of hepatic steatosis and liver fibrosis in NAFLD. Larger prospective studies with long-term follow up are warranted to provide more definitive evidence. Second, liver biopsy remains the gold standard for the detection of liver steatosis and fibrosis, but is invasive, impractical and costly. We used noninvasive ultrasonography examination and transient elastography for noninvasive assessment of hepatic steatosis and fibrosis. Third, as levels of anti-thyroid peroxidase and anti-thyroglobulin autoantibodies were not measured in our study, we cannot determine the possible role of impending thyroid autoimmunity on the association between NAFLD with thyroid function.

## Conclusions

There are positive associations of FT3 levels with the severity of hepatic steatosis and liver fibrosis in euthyroid NAFLD subjects. Additional large-scaled prospective studies should be undertaken to confirm these findings. Further research is needed to determine whether these findings are causal, and whether the FT3 levels have any role as biomarkers for NAFLD.

## Data Availability Statement

The original contributions presented in the study are included in the article/supplementary material. Further inquiries can be directed to the corresponding author.

## Ethics Statement 

The studies involving human participants were reviewed and approved by Human Research Ethics Committee of the First Affiliated Hospital of Nanjing Medical University. The patients/participants provided their written informed consent to participate in this study.

## Author Contributions

WG, and QZ participated in the study design. WG, PQ, X-NL, JW, JL, W-FZ, Q-qD, and N-ZX were involved in the conduct of the study and data collection. WG and PQ made contributions to the data analysis and interpretation of the results. WG and QZ wrote and modified the manuscript and prepared the tables and figures. All authors contributed to the article and approved the submitted version.

## Funding

Financial support for this project was from The Natural Science Foundation of the Jiangsu Higher Education Institutions of China (20KJB320009) and Science and Technology Department of Jiangsu Province (No. BE2016787).

## Conflict of Interest

The authors declare that the research was conducted in the absence of any commercial or financial relationships that could be construed as a potential conflict of interest.

## Publisher’s Note

All claims expressed in this article are solely those of the authors and do not necessarily represent those of their affiliated organizations, or those of the publisher, the editors and the reviewers. Any product that may be evaluated in this article, or claim that may be made by its manufacturer, is not guaranteed or endorsed by the publisher.

## Supplementary Material

Supplementary Figure 1 The association of FT3 levels with FAP value and LSM value. **(A)**. The association of FT3 levels with FAP value in all participants; **(B)**. The association of FT3 levels with LSM value in participants with NAFLD).
