# Supplementary material for: Free Triiodothyronine Is Associated With Hepatic Steatosis and Liver Stiffness in Euthyroid Chinese Adults With Non-Alcoholic Fatty Liver Disease
Source: Front Endocrinol (Lausanne). 2021 Aug 12;12:711956. doi: 10.3389/fendo.2021.711956 (PMC8387962; doi:10.3389/fendo.2021.711956)
Supplement: Supplementary Figure 1 — The association of FT3 levels with FAP value and LSM value. (A). The association of FT3 levels with FAP value in all participants; (B). The association of FT3 levels with LSM value in participants with NAFLD). [file Image_1.pdf]

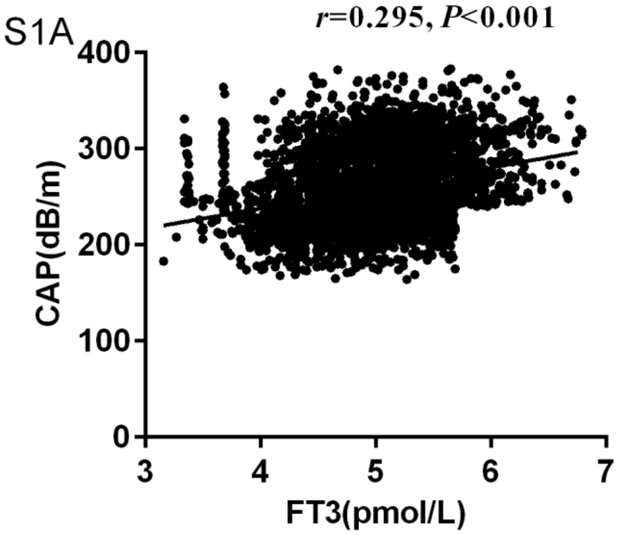

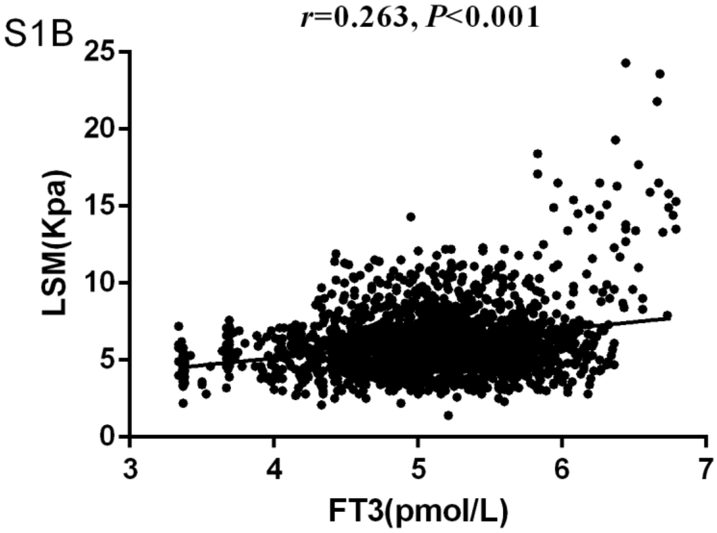

Supplement Figure 1. The association of FT3 levels with FAP value and LSM value. (S1A. The association of FT3 levels with FAP value in all participants; S1B. The association of FT3 levels with LSM value in participants with NAFLD)
